# Mapping the nomenclature, methodology, and reporting of studies that review methods: a pilot methodological review

**DOI:** 10.1186/s40814-019-0544-0

**Published:** 2020-01-30

**Authors:** Daeria O. Lawson, Alvin Leenus, Lawrence Mbuagbaw

**Affiliations:** 1 Department of Health Research Methods, Evidence, and Impact, McMaster University, 1280 Main Street West, Hamilton, ON L8S 4K1 Canada; 2 Faculty of Health Sciences, McMaster University, 1280 Main Street West, Hamilton, ON L8S 4K1 Canada; 3 Biostatistics Unit, Father Sean O’Sullivan Research Centre, St. Joseph’s Healthcare Hamilton, Hamilton, ON L8N 4A6 Canada

**Keywords:** Feasibility, Guidelines, Methodological review, Nomenclature, Pilot, Reporting

## Abstract

**Background:**

A relatively novel method of appraisal, methodological reviews (MRs) are used to synthesize information on the methods used in health research. There are currently no guidelines available to inform the reporting of MRs.

**Objectives:**

This pilot review aimed to determine the feasibility of a full review and the need for reporting guidance for methodological reviews.

**Methods:**

Search strategy: We conducted a search of PubMed, restricted to 2017 to include the most recently published studies, using different search terms often used to describe methodological reviews: “literature survey” OR “meta-epidemiologic* review” OR “meta-epidemiologic* survey” OR “methodologic* review” OR “methodologic* survey” OR “systematic survey.”

Data extraction: Study characteristics including country, nomenclature, number of included studies, search strategy, a priori protocol use, and sampling methods were extracted in duplicate and summarized.

Outcomes: Primary feasibility outcomes were the sensitivity and specificity of the search terms (criteria for success of feasibility set at sensitivity and specificity of ≥ 70%).

Analysis: The estimates are reported as a point estimate (95% confidence interval).

**Results:**

Two hundred thirty-six articles were retrieved and 31 were included in the final analysis. The most accurate search term was “meta-epidemiological” (sensitivity [Sn] 48.39; 95% CI 31.97–65.16; specificity [Sp] 97.56; 94.42–98.95). The majority of studies were published by authors from Canada (*n* = 12, 38.7%), and Japan and USA (*n* = 4, 12.9% each). The median (interquartile range [IQR]) number of included studies in the MRs was 77 (13–1127). Reporting of a search strategy was done in most studies (*n* = 23, 74.2%). The use of a pre-published protocol (*n* = 7, 22.6%) or a justifiable sampling method (*n* = 5, 16.1%) occurred rarely.

**Conclusions:**

Using the MR nomenclature identified, it is feasible to build a comprehensive search strategy and conduct a full review. Given the variation in reporting practices and nomenclature attributed to MRs, there is a need for guidance on standardized and transparent reporting of MRs. Future guideline development would likely include stakeholders from Canada, USA, and Japan.

## Background

Health researchers, methodologists, and policymakers rely on primary studies or evidence syntheses (e.g., systematic reviews) to provide summaries of evidence for decision-making [1, 2]. However, the credibility of this evidence depends on how the studies were conducted and reported. Therefore, critical appraisal of health research methodology is an important tool for researchers and end-users of evidence. As such, certain studies exist solely to help synthesize methodological data about the design, analysis, and reporting of primary and secondary research. These studies can be referred to, for the purposes of this paper, as methodological reviews (MRs) and represent an efficient way of assessing research methods and summarizing methodological issues in the conduct, analysis, and reporting of health research. Collating primary and secondary research in this way can help to identify reporting and methodological gaps, generate empirical evidence on the state of or quality of conduct and reporting, and inform the development of reporting and methodological standards. MRs are highly informative because they allow researchers to evaluate study methods; assess adherence, quality, and completeness of reporting (e.g., reporting adherence to Consolidated Standards of Reporting Trials, CONSORT); document and assess the variety of methods used or approaches to analyses (e.g., statistical approaches for handling missing data in cluster randomized trials); demonstrate changes in reporting over time (e.g., since the introduction of a specific guideline); demonstrate consistency between study abstracts/trial registries and their full texts; and many other issues [3–8]. In this way, MRs are indispensable to high-quality health research by allowing researchers to identify inappropriate research method practices and propose solutions.

Reporting guidelines are important tools in improving the reporting and conduct of health research, and many exist for various study designs. Currently, the Enhancing the QUAlity and Transparency Of health Research (EQUATOR) network is the leading authority in reporting guidelines for health research [9]. As of September 2019, this website lists 418 guidelines, with another 74 currently under development. There is empirical evidence that publication of reporting guidelines improves reporting, but this is often contingent on journal endorsement as well as the period of time since publication [10–12]. However, there is no published guidance for reporting methodological reviews despite an increase in their development and usage [13].

Murad and Wang have proposed a checklist which is an adaptation of the Preferred Reporting Items for Systematic Reviews and Meta-Analyses (PRISMA), a widely endorsed guideline developed to help report systematic reviews [14, 15]. While its use would ensure that a standardized and transparent approach in reporting is followed, this does not address the various typologies of MRs including the variety of approaches used to conduct these studies. For example, MRs that use a before-after design, interrupted time series, or random sampling approaches would be a poor fit for this tool [7, 16–18]. Likewise, studies in which the unit of analysis is not the “study” would require more specific guidance (e.g., some MRs investigate multiple subgroup analyses within the same study) [18, 19]. Further, some MRs report formal sample size estimations, and it is unclear whether this should be recommended for all MRs. In line with this thinking, recent correspondences with the Journal of Clinical Epidemiology highlight that methodological studies cannot always be classified as systematic reviews, but instead represent their own branch of evidence synthesis methodology requiring specially tailored reporting guidance [20].

To facilitate the development of reporting guidelines for health research, Moher et al. propose a five-phase, 18-step strategy [10]. One important step in this process is a review of the literature, including seeking evidence on the quality of published research articles and identifying information related to sources of bias in reporting. However, some concerns exist with reviewing the literature. First, the literature on methodological reviews is elusive and can be found in any journal or database. Second, given the relative novelty and rapid development of the field, there is no formally accepted nomenclature to guide a literature search. We therefore deemed it necessary to conduct a pilot methodological review of methodological reviews to inform the feasibility of a broader, full methodological review based on our ability to

1. Determine the appropriate nomenclature for identification of methodological reviews
2. Determine a preliminary need for guidance, based on inconsistencies in reporting

Pilot studies of research syntheses, contrary to pilot studies of classical “primary” research, aim to: establish the need for a full review, establish the value of the methods used, and to identify, clarify, and review any problems with the processes and instruments. They can also be used to identify conceptual, methodological, and practical problems that need to be addressed in a full review. In this way, piloting research syntheses maximizes validity and efficiency [21, 22]. The research questions we sought to answer in this pilot review, where they fit in the larger scheme of the project objectives, and their implications for a full review (i.e., larger study to further explore the observed methodological variations in a broader sample of MRs) are outlined in Table 1. The listed research questions were evaluated in and applied to the MRs included in this pilot.

**Table 1:** Pilot research questions and implications for a full review

Pilot review objectives	Research questions	Implications for feasibility of full review	Metrics/threshold
Determine the appropriate nomenclature for accurate identification of methodological reviews	Which search terms yield methodological reviews?	Identifying a list of terms that yields methodological reviews will inform the search strategy in the full review	Sensitivity/specificity ≥ 70%
Determine the need for methodological review reporting guidelines	Are research methods specified a priori?	Inconsistent pre-specification of methods would indicate the need for a full review	≤ 70% with published protocols
	How many databases are searched?	Wide variation in the numbers of databases searched would indicate the need for a full review	Coefficient of variation ~ 1 (i.e., spread in results relative to the mean)
	Are search time limits justified?	Inappropriate justification of search time limits would imply the need for a full review	≤ 70% justify search limits
	Is the sample size justified?	Inappropriate justification of sample size for MRs designed as analytical studies (e.g., before-after comparisons, regression-based analyses) would imply the need for a full review	≤ 70% justify sample size or perform sample size calculation
	Is a formal sample size calculation performed?	Inappropriate justification of sample size for MRs designed as analytical studies (e.g., before-after comparisons, regression-based analyses) would imply the need for a full review	
	Is a random sample of studies used?	Use of different sampling approaches to select a subset of studies from a larger group would indicate the need for a full review	Among studies where the goal was not to capture all available studies, ≤ 70% use a random sampling approach
	Do research methods or authors suggest generalizable findings?	Lack of clear approaches to reporting generalizability would indicate the need for a full review	≤ 70% discuss the generalizability of findings

## Methods

### Study design

The methods reported are in line with current guidance on piloting evidence syntheses, by way of a “mini-review” all the way through [21]. We conducted a pilot methodological review of a sample of methodological reviews published in 2017.

### Eligibility criteria

We included articles that fulfilled all of the following criteria:

- Are in the domain of clinical research with human participants
- Could be classified as secondary research (i.e., investigating other studies/primary research)
- Investigate methods or reporting issues

### Search strategy

We conducted a search of PubMed—a public search engine which retrieves medical literature from the MEDLINE database—from January 1, 2017 to December 31, 2017 (i.e., the most recent complete year) using terms often used to refer to MRs: “literature survey” OR “meta-epidemiologic* review” OR “meta-epidemiologic* survey” OR “methodologic* review” OR “methodologic* survey” OR “systematic survey”. To maintain a focused search as was intended for the scope of this pilot, phrase searching was used to restrict the volume of hits with wildcard searching.

### Study selection

One reviewer (DOL) screened the titles and abstracts of retrieved articles in a reference manager program (EndNote X7.8, Philadelphia: Clarivate Analytics; 2016) for the type of study and whether any nomenclature was present in either or both the title and abstract. Studies identified as methodological reviews were screened in duplicate (DOL and AL) and verified for eligibility in the full texts using a spreadsheet (Microsoft Excel for Mac v.16.15, Washington: Microsoft Corporation; 2018).

### Data extraction

Two reviewers (DOL and AL) extracted data from MRs in a spreadsheet in duplicate including the first author name, country of primary affiliation (for > 1 co-first authors, the affiliation of the first listed author was taken; for > 1 affiliations of the first author, the senior author’s affiliation was taken), nomenclature in the title, nomenclature in the abstract, nomenclature in the methods section, total number of included records (e.g., abstracts, instruments, journals, meta-analyses, reviews, and trials), if the sample size was calculated, if the authors referenced a published protocol, the databases searched for record inclusion, if there was justification of search time limits, whether the search strategy was reported (or referenced elsewhere), and if sampling of records was random.

Given our concerns with sampling issues, we sought additional information on generalizability. Generalizability was guided by the response to the following question: “Do these findings represent the total population of studies that the sample was drawn from?”. We classified studies as likely generalizable if they met some or all of the following criteria:

- Used multiple databases
- Justified their sample size (e.g., provide details for a sample size calculation)
- Selected a random sample of records (where applicable)

We classified studies as unlikely to be generalizable if they

- Used only one database
- Used selected journals (e.g., only high impact)
- Used very stringent eligibility criteria

Disagreements were resolved through discussion, and if reviewers could not come to an agreement on conflicts, a third reviewer (LM) adjudicated as necessary.

### Inter-rater agreement

We assessed the level of agreement between reviewers using Cohen’s kappa (*κ*) for inter-rater reliability for two raters. Agreement was calculated for yes/no and numerical fields at the full-text screen and data extraction levels. The index value was interpreted as no agreement (0–0.20), minimal agreement (0.21–0. 39), weak agreement (0.40–0.59), moderate agreement (0.60–0.79), strong agreement (0.80–0.90), and almost perfect agreement (above 0.90) [23].

### Data analysis

We summarized and reported the descriptive statistics, including frequencies and percentages for count and categorical variables, and means with standard deviation (SD) for continuous variables.

In order to determine the best search strategy, we computed the sensitivity and specificity of each search term to determine which would have the best accuracy in our database of studies identified from our search. We computed the proportion of studies identified with each search term that were actually MRs (i.e., included studies and true positives) and the proportion identified that were not MRs (i.e., excluded studies and false positives). We also computed the proportion of studies not captured by the search term that were not MRs (i.e., excluded studies and true negatives) and the proportion of MRs that were not captured by the search term (i.e., included studies and false negatives). We pooled the sensitivity and specificity estimates for multiple search terms using the parallel testing approach in order to achieve an optimal combination of search terms with a good balance of sensitivity and specificity [24]. Statistical analysis was conducted in IBM SPSS Statistics (IBM SPSS Statistics v.24.0, Armonk: IBM Corporation; 2016) and inter-rater agreement (*κ* with 95% confidence interval, CI) was calculated using WinPepi [25]. We built a word cloud in WordArt to visualize common terms used for indexing MRs in PubMed [26].

### Ethics review

Ethics committee approval and consent to participate was not required as this study used previously published non-human data.

## Results

**Fig. 1:**
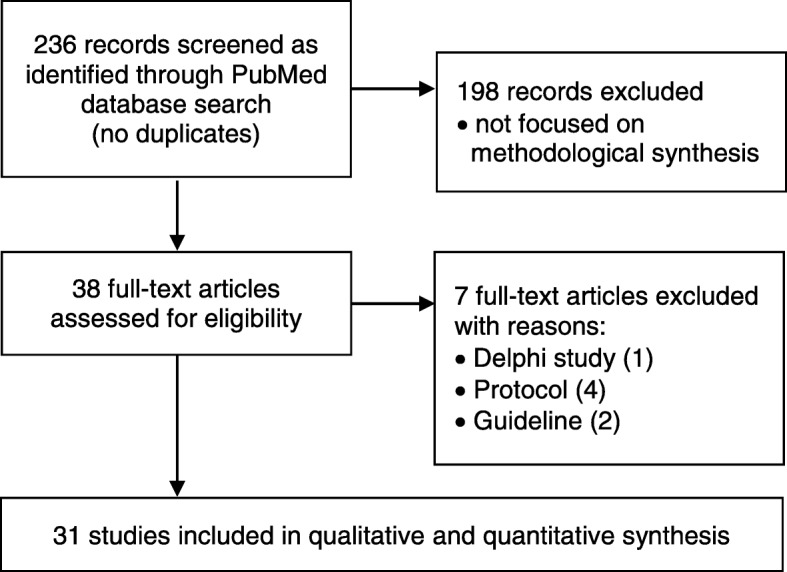
Study flow diagram illustrating selection of eligible studies

There were 236 articles retrieved from the PubMed search of which 31 were included in the final quantitative and qualitative analysis (see Fig. 1 for study flow diagram with reasons for exclusion) [27–57]. There was moderate inter-rater agreement between reviewers before consensus (*κ* = 0.78; 95% CI 0.73–0.82).

The study characteristics of all included studies are outlined in the Appendix.

### Characteristics of included methodological reviews

Many of the authors were from Canadian institutions (*n* = 12, 38.7%), followed by Japan and USA (*n* = 4, 12.9% each). Based on the previously defined criteria, we scored ten studies (32.3%) as generalizable [28, 30, 38, 40, 49, 51–54, 57]. Only three studies (9.7%, two of which we scored as generalizable) commented on generalizability and reported their own work as generalizable, either to the subject area (e.g., venous ulcer disease), to a clinical area, or in general terms [27, 30, 38].

### Nomenclature

**Fig. 2:**
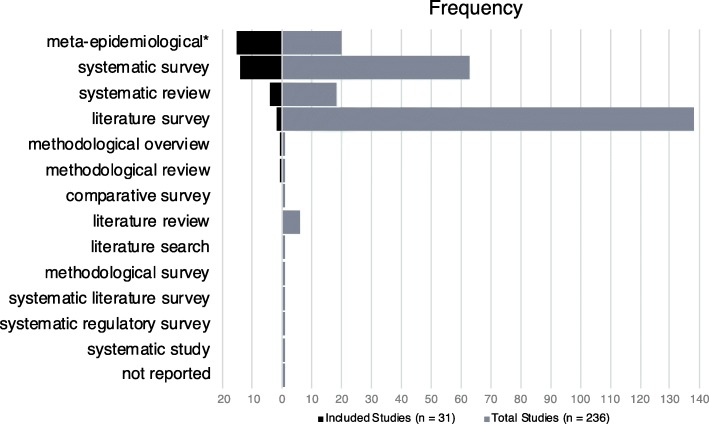
Pyramid graph illustrating a comparison of nomenclature from PubMed search and included studies. Frequencies are based on terms as reported in the study title and/or abstract, and taking into account studies that used more than one term (i.e., total terms for included studies *n* = 37 and total terms for all studies from PubMed search *n* = 254). "Meta-epidemiological" includes all nine variants that were captured by the search term “meta-epidemiologic*”

Of the 31 included studies, 77.4% (*n* = 24) presented the study nomenclature in their title. The terms found in the titles and abstracts of all retrieved articles that were used to describe the study are represented in Fig. 2. The most accurate search terms were “meta-epidemiological” (sensitivity [Sn] 48.39; 95% CI 31.97–65.16; specificity [Sp] 97.56; 94.42–98.95), “systematic survey” (Sn 45.16; 95% CI 29.16–62.23; Sp 76.10; 95% CI 69.81–81.42) “systematic review” (Sn 12.90; 95% CI 5.13–28.85; Sp 93.17; 95% CI 88.86–95.89), and “literature survey” (Sn 6.45; 95 CI 1.79–20.72; Sp 33.66; 95% CI 27.54–40.38), among 12 different types or combinations of nomenclature cited (Fig. 2). The combined sensitivity and specificity for the six terms attributed to MRs was 100% and 0.99%, respectively.

The words “survey” and “systematic” (*n* = 18 each), “meta-epidemiologic*” (*n* = 15), and “review” and “study” (*n* = 7 each) were the most frequent in the word cloud (Appendix). Five (16.1%) of these studies used more than one name to describe their study type in the title or abstract (two names, *n* = 4, and three names, *n* = 1).

### Methodological features

The mean (standard deviation [SD]) number of databases searched was 2 (1.6) with a minimum of 1 and a maximum of 8 databases. Overall, less than a quarter of studies (*n* = 7, 22.6%) made a reference to or cited a protocol for the study. Most studies reported the search strategy (*n* = 23, 74.2%) and only eight (25.8%) justified the time limits that were set for the search (Table 2).

**Table 2:** Methodological features of included methodological reviews (*N* = 31)

Variable	*n* (%)
Reported study type (nomenclature) in the “Methods” section	12 (38.7)
Number of databases searched (mean, SD)	2 (1.6)
Number of records included (median, IQR)	77 (13 – 1127)
Reported (or referenced) a protocol	7 (22.6)
Reported (or referenced) a search strategy	23 (74.2)
Justified search time limits	8 (25.8)
Performed a sample size calculation a priori	5 (16.1)
Randomly sampled included records (of *n* = 5)	5 (100)

*IQR* interquartile range, *SD* standard deviation

Five studies (16.1%) performed an a priori sample size calculation of records to be included, using a variety of methods. Abbade et al. used an estimate for the primary objective from a prior similar study [27], and similarly, Riado Minguez et al. used a prior similar study to determine a target sample size coupled with a power calculation [43]. El Dib et al. sampled enough studies to achieve a CI of ± 0.10 around all proportions [33], and Zhang et al. used a precise CI of ± 0.05 [57]. Kosa et al. incorporated an approach optimized for logistic regression, based on estimates for correlation between covariates [37]. Among the studies that did not aim to summarize data from all available records retrieved in their search (*n* = 5), all studies (100%) incorporated some randomization strategy to sample the records to be included in their final synthesis [29, 33, 34, 37, 57].

## Discussion

**Fig. 3:**
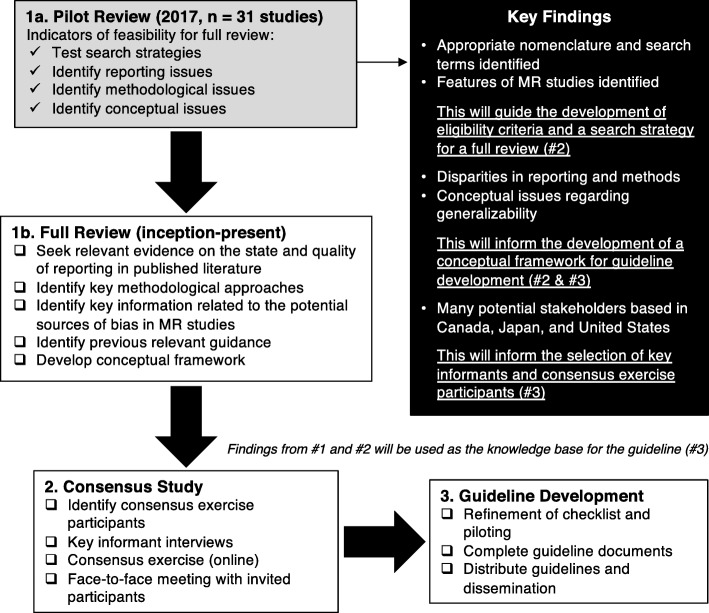
Stages of development of reporting guidelines for methodological reviews

In this pilot methodological review, we have established the need for a full review and determined some of the methodological features worth investigating to facilitate the development of a reporting guideline for MRs. Unquestionably, it is highly likely that our search strategy missed MRs characterized by different nomenclature. However, as a result of this pilot we have been able to identify some of the most appropriate search terms to incorporate into a search strategy in the full review. The criteria for success of feasibility and the respective results are outlined in Table 3. The position of this pilot in the larger picture of the development of the reporting guideline is outlined in Fig. 3. Additionally, the forthcoming guideline as a result of this work, the METhodological Review reportIng Checklist (METRIC), has been registered as currently under development with EQUATOR.

**Table 3:** Feasibility results for this pilot review

Measure	Target	Observed	Description
Sensitivity/specificity^b^	≥ 70%	Sensitivity, 100% Specificity, 0.99%	Six terms combined gave good sensitivity but compromised specificity
Published protocols^a^	≤ 70%	22.6%	Few studies had pre-specified methods
Coefficient of variation^c^	~ 1	0.8	Fairly consistent number of databases searched
Justification of search limits^a^	≤ 70%	25.8%	Few studies justified their search limits
Justification of sample size or perform sample size calculation^a^	≤ 70%	16.1%	Few studies justified their sample sizes or performed calculations
Use a random sampling approach^c^	≤ 70%	100%	All studies adopted a random sampling approach
Discuss the generalizability of findings^a^	≤ 70%	9.7%	Few studies described how generalizable their findings were

^a^Feasibility criteria met

^b^Feasibility criteria partially met

^c^Feasibility criteria not met

The disparity in nomenclature, methods, and reporting in this assessment of 1-year worth of data suggests that a full review is required to better provide a more complete picture of the existing concerns with reporting quality. A number of key features stand out and an appraisal of these concerns would also help to inform the development of guidance. First, there is a growing body of literature pertaining to reviews of methods. A search of PubMed with the term “methodological review” shows that there has been a steady increase in studies indexed as MRs over the past 10 years, with ten in 2007 and 39 in 2017. The increasing number of publications addressing methodological issues in primary and secondary research would suggest that there is interest in understanding and optimizing health research methods. Therefore, the development of consensus-based guidelines in this field is warranted.

Second, there are inconsistencies in the nomenclature used to describe MRs. In the variety of names currently being attributed to MRs, nomenclature is an issue that must be addressed. This is especially true with the use of labels such as “systematic review”—which is attributed to a specific, well-defined form of evidence synthesis for healthcare studies—or in the case of some studies which have used the term “methodologic,” which is not otherwise defined in English dictionaries and which could compromise their detection in searches [58].

Third, there are no methodological standards specific to MRs. Regarding selection bias, it is unclear what processes researchers employ in defining the appropriate eligibility criteria, or in the selection of databases and time periods for screening relevant literature. Readers would be interested in knowing why and how the choice of studies is ideal to answer the research question and the rationale behind such choices should be explicit. Likewise, the approaches to the sampling should be explicit, especially in methodological reviews that do not adopt systematic searches to identify and capture all of the relevant articles. For example, some methodological studies might approach a research question with a before-after, cross-sectional, or longitudinal design to name a few. As a result, it may not be appropriate or necessary to use all of the available studies in these scenarios and a sample of studies may suffice [59, 60].

On a conceptual level, we sought to develop a definition of study generalizability, but this was challenging to operationalize. The aim of this exercise was to help define the scope of inferences that can be made from the findings in MRs. As this area currently lacks specific guidance, we recognize that the appropriateness of the selected criteria (and its applicability to each study) will see ongoing development in subsequent investigations and may be applied differently. Generalizability is strongly tied to the target population and this was not often explicit in MRs, making any inferences challenging. Our approach to defining generalizability could be refined with insights from authors and users of MRs and will vary based on the study in question. There are several ways of addressing this outcome: do the authors identify their study as generalizable? Is the “study topic” (i.e., methodological issue) generalizable to other fields? Are the “results” generalizable to other studies in different fields investigating the same methodological issue? How are these results applicable?

Conversely, we can also consider whether the sample size and number of databases searched are surrogate indicators of generalizability. These factors could be used to extrapolate to the generalizability of an MR, as is done with clinical trials and systematic reviews, and as we have done in the present study. These questions and the importance of this variable might be answered with a deeper investigation of MRs, as well as feedback and engagement from expert users as we work to develop guidance on reporting. Through expert consensus, and recognizing typological differences, we also plan to optimize the proposed guidance for specific types of MRs (e.g., MRs assessing methods of randomized control trials or systematic reviews). We hope that these approaches will also help to tease out the appropriate definition of generalizability in each case.

## Conclusions

We now have a clearer understanding of the terms used to describe methodological reviews and some of the issues that warrant a deeper investigation. In this pilot review, we have highlighted the need for a full review on this topic in order to inform future guidance for reporting methodological reviews. A full review using some of the search terms identified here is feasible. These findings will be used to develop a protocol, which will encompass more databases and years, in order to gain a clearer sense of the landscape of MRs.

## Figures and Tables

**Fig. 4:**
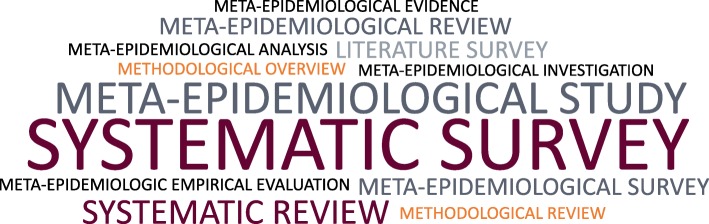
Word cloud depicting frequency of PubMed MeSH terms used to describe and catalog methodological studies

## Data Availability

In addition to the supplementary information files which are included in this published article, the dataset generated and analyzed during the current study is available from the corresponding author on reasonable request.

## Appendix

**Table 4:** Main characteristics of included methodological reviews (*n* = 31)

Study	Country	Nomenclature (T/A)	Nomenclature (M)	Databases searched (#)	Records included (#)	Reference to a protocol (Y/N)	Search strategy reported (Y/N)	Justification of search time limits (Y/N)	Sample size calculation (Y/N)	Random sampling (Y/N)
Abbade et al. [27]	Canada	SSu	SSu	1	85	Y	Y	Y	Y (61)	N
Abdul-Khalek et al. [28]	Lebanon	SSu	None	2	57	N	Y	N	N	N
Armijo-Olivo et al. [29]	Canada	MES	MES	1	393	Y	Y	N	N	Y
Bolvig et al. [30]	Denmark	MES	None	1	126	Y	Y	N	N	N
Chase Kruse and Matt Vassar [31]	USA	SSu	SSu	2	35	N	N	N	N	N
Ebrahim et al. [32]	Canada	SR/SSu	None	3	28	Y	Y	N	N	N
El Dib et al. [33]	Canada	SSu	None	1	103	N	Y	N	Y (100)	Y
Ge et al. [34]	China	MES	MESc	1	150	N	Y	N	N	Y
Gorne and Diaz [35]	Argentina	SR/SSu	LSu	1	128	N	Y	N	N	N
Khan et al. [36]	Canada	SSu	SSu	3	48	N	Y	N	N	N
Kosa et al. [37]	Canada	MR, SSu	MR	1	200	N	Y	Y	Y (152)	Y
Kovic et al. [38]	Canada	MESu	MES, SR	2	77	N	Y	Y	N	N
Kuriyama et al. [39]	Japan	LSu	None	1	353	N	N	Y	N	N
Manja et al. [40]	Canada	SSu	None	5	43	N	Y	N	N	N
Papageorgiou et al. [41]	Switzerland	MEE, MO, SR	SR	8	34	Y	Y	N	N	N
Ratib et al. [42]	UK	MER	None	1	1127	N	N	N	N	N
Riado Minguez et al. [43]	Spain	MES	None	1	446	N	Y	Y	Y (150)	N
Sekercioglu et al. [44]	Canada	SSu	None	5	16	N	Y	N	N	N
Shinohara et al. [45]	Japan	MEI, SR	None	1	60	Y	Y	N	N	N
Sims et al. [46]	USA	MES	MES, Su	1	37	Y	N	N	N	N
Storz-Pfennig [47]	Germany	MEA	None	1	13	N	N	N	N	N
Tedesco et al. [48]	Italy	MEEE	None	1	244	N	N	N	N	N
Tsujimoto et al. [49]	Japan	MES	None	3	326	N	N	Y	N	N
Tsujimoto et al. [50]	Japan	MES	None	1	284	N	Y	Y	N	N
Umberham et al. [51]	USA	MER	None	2	265	N	Y	N	N	N
von Niederhausern et al. [52]	Switzerland	SSu	None	2	47	N	Y	N	N	N
Wallach et al. [53]	USA	MESu	MESu	3	64	N	N	N	N	N
Yepes-Nunez et al. [54]	Canada	SSu	None	3	42	N	Y	N	N	N
Yu et al. [55]	Taiwan	LSu	LSu of SRs	1	29	N	Y	N	N	N
Zhang et al. [56]	Canada	SSu	None	4	60	N	Y	N	N	N
Zhang et al. [57]	Canada	SSu	None	1	200	N	Y	Y	Y (200)	Y

*LSu* literature survey, *M* methods section, *MEA* meta-epidemiological analysis, *MEE* meta-epidemiological evidence, *MEEE* meta-epidemiologic empirical evaluation, *MEI* meta-epidemiological investigation, *MER* meta-epidemiological review, *MES* meta-epidemiological study, *MESc* comparative meta-epidemiological study, *MESu* meta-epidemiological survey, *MO* methodological overview, *MR* methodological review, *N* no, *SR* systematic review, *SSu* systematic survey, *Su* survey, *T/A* title or abstract section, *UK* United Kingdom, *USA* United States of America, *Y* yes

## Abbreviations

CI: Confidence interval
CONSORT: Consolidated Standards of Reporting Trials
EQUATOR: Enhancing the QUAlity and Transparency Of health Research
IQR: Interquartile range
MeSH: Medical Subject Headings
MR: Methodological review or review of methods
*N*/*n*: Sample size
PRISMA: Preferred Reporting Items for Systematic Reviews and Meta-Analyses
SD: Standard deviation
